# International, multi-disciplinary, cross-section study of pain knowledge and attitudes in nursing, midwifery and allied health professions students

**DOI:** 10.1186/s12909-022-03488-3

**Published:** 2022-07-15

**Authors:** Jagjit Mankelow, Cormac G. Ryan, Paul C. Taylor, Maire-Brid Casey, Jenni Naisby, Kate Thompson, Joseph G. McVeigh, Chris Seenan, Kay Cooper, Paul Hendrick, Donna Brown, William Gibson, Mervyn Travers, Norelee Kennedy, Cliona O’Riordan, Denis Martin

**Affiliations:** 1 Teesside University, Middlesbrough, England; 2 University of Limerick, Limerick, Ireland; 3 University College Dublin, Dublin, Ireland; 4 Northumbria University, Northumbria, England; 5 Leeds Beckett University, Leeds, England; 6 University College Cork, Cork, Ireland; 7 Glasgow Caledonian University, Glasgow, Scotland; 8 Robert Gordon University, Aberdeen, Scotland; 9 University of Nottingham, Nottingham, England; 10 University of Ulster, Belfast, Northern Ireland; 11 The University of Notre Dame Australia, Fremantle, Australia; 12 Curtin University, Perth, Australia; 13 Applied Research Collaboration for the North East and North Cumbria, National Institute for Health and Care Research, Newcastle Upon Tyne, England

**Keywords:** Pain education, Healthcare students, Cross-section

## Abstract

**Background:**

Persistent pain is a highly prevalent, global cause of disability. Research suggests that many healthcare professionals are not well equipped to manage pain, and this may be attributable at least in part to undergraduate education. The primary aim of this study was to quantify and compare first and final year nursing, midwifery and allied health professional (NMAHP) students’ pain related knowledge and attitudes. The secondary aim was to explore what factors influence students’ pain related knowledge and attitudes.

**Methods:**

In this cross-sectional study, 1154 first and final year healthcare students, from 12 universities in five different countries completed the Revised Neurophysiology of Pain Quiz (RNPQ) [knowledge] and the Health Care Providers Pain and Impairment Relationship Scale (HC-PAIRS) [attitudes].

**Results:**

Physiotherapy was the only student group with statistically and clinically improved pain related knowledge [mean difference, 95% CI] (3.4, 3.0 to 3.9, *p* = 0.01) and attitudes (-17.2, -19.2 to 15.2, *p* = 0.01) between first and final year. Pain education teaching varied considerably from course to course (0 to 40 h), with greater levels of pain related knowledge and attitudes associated with higher volumes of pain specific teaching.

**Conclusions:**

There was little difference in pain knowledge and attitudes between all first and final year NMAHP students other than physiotherapy. This suggests that for most NMAHP disciplines, undergraduate teaching has little or no impact on students’ understanding of pain. There is an urgent need to enhance pain education provision at the undergraduate level in NMAHPs.

**Trial Registration:**

The study protocol was prospectively registered at ClinicalTrials.Gov NCT03522857.

## Background

Pain is amongst the most common reason patients engage with health care [1–3]. Pain, the unpleasant sensory and emotional experience associated with actual or potential tissue damage, can be classified by duration of symptoms as acute, sub-acute or chronic pain [4, 5]. High rates of pain are present globally. For example, chronic pain affects 28 million people in the UK alone [6] and is often associated with significant disability [7]. Similarly, over three million Australians identify as living with chronic pain. The economic burden amounts to AUD 73.2 billion each year including AUD 48.3 billion in lost productivity [8]. But the issue cannot be adequately captured by dollars lost. Chronic pain negatively affects quality of life affecting physical, mental, and social health [9]. The Prevalence Impact and Cost of Chronic Pain (PRIME) study conducted in Ireland reported a chronic pain prevalence rate of 35.5%. Over 37% of those with pain reported moderate to severe pain-related disability [10].

Multiple disciplines are involved in the management of pain, therefore it is vital that all health care professionals (HCP) in every health care discipline are well equipped to manage this problem and have a good knowledge of pain and positive attitudes towards function in those with pain. Furthermore, it is imperative that this management is evidence-based and guideline-compliant to ensure consistent high-quality care which is individualised [11, 12].

Existing research suggests that many HCPs across the disciplines are not well equipped to manage pain. Non-evidence based and inconsistent patterns of pain management occur frequently in various health care settings which results in the high use of resources [13–16]. Clinicians often do not feel confident or able to treat patients with persistent pain [17–19]. Furthermore, there is evidence to suggest that HCPs’ attitudes about the functional ability of people in pain influences their management recommendations, and this in turn influences patients’ attitudes about pain and their health outcomes [20–24]. Patients often have a biomedical understanding of their pain and link it to structural damage. These attitudes seem to be influenced by their HCPs’ pain knowledge and attitudes which are often also biomedical [25, 26]. It is important that HCPs’ pain attitudes and knowledge are evidence-based [12]. However, it is widely recognised that this is not always the case. It has been suggested that a part of this problem may be the absence of adequate pain education in pre-registration training [27–29]. Knowledge is accepted as a component of attitudes, which are key indicators of behaviour [30]. It has been proposed that improved understanding of pain amongst clinicians would improve the delivery of evidence-based care, leading to better patient outcomes [31].

The inadequacy of pain education in health care curricula has been observed throughout Europe, New Zealand and Australia, the USA and Canada [32–35]. The first step towards addressing the deficiency in pain education among HCPs would be to assess current pain understanding amongst HCP students. A number of studies have explored this issue, however, these studies are generally limited to single institutions, discrete regions or only a small number of health care disciplines, reducing the generalisability of the findings [36–40]. If some disciplines were found to have poorer pain-related understanding than others, this difference could be explored, and pain education resources could be targeted accordingly.

The primary aim of this study was to quantify and compare nursing, midwifery and allied health professional (NMAHP) students’ knowledge and attitudes about pain management in the first and the final year of their studies across a range of disciplines in multiple institutions and countries. The secondary aim was to explore some of the factors that may influence students’ pain related knowledge and attitudes towards the functional ability of people with pain.

## Method

### Design

In this observational, cross-sectional study the attitudes and knowledge of first and final year NMAHP students were collected using two questionnaires to establish the change during undergraduate health care degree courses. The attitudes and knowledge of students were compared. The questionnaires were administered in the first semester for first years and as close as possible to the completion of the degree course in the case of final year students. Data on participants’ age, gender, and year of study and course of study were collected.

### Ethics

Ethical approval for this study was initially granted by Teesside University’s (TU) School of Health and Social Care Research Ethics and Governance Committee local ethics project number 114/17. Each of the other eleven collaborating Universities obtained permission from their respective University’s research ethics and governance committee. The study protocol was prospectively registered at ClinicalTrials.Gov NCT03522857, https://clinicaltrials.gov/ct2/show/ NCT03522857.

### Participants and recruitment

First year and final year BSc and MSc pre-registration students were recruited between the period of October 2017 to September 2019, from 12 universities and six disciplines across Australia, England, Northern Ireland, the Republic of Ireland and Scotland. NMAHP disciplines were selected based on those frequently involved in pain management, and included physiotherapy, occupational therapy, paramedics, diagnostic radiography, midwifery, and nursing. To meet the inclusion criteria for participation, individual students needed to be in the first or final year of their studies within one of the aforementioned disciplines.

Collaborating universities were invited to take part through informal networks, via on-site academics acting as local pain education “champions”. Pain champions disseminated the recruitment invitation to local programme leaders for delivery to students and either disseminated and collected surveys physically or directed students to the online survey. A reminder email was sent two weeks later. Additionally, where possible, the local champions delivered short presentations to student groups to raise awareness of the study. Paper questionnaires were made available at these presentations and a confidential drop box at a different location from the distribution site was provided for questionnaire collection. The site of questionnaire distribution and collection were kept separate in order to ensure that students did not feel obliged to participate in the study. Participants were asked to complete the survey only once when they received a reminder email. The participant information sheet explained to prospective participants that consent was implied by completion of the survey.

Participating universities were invited to provide information about the extent and format of pain education within the disciplines surveyed. Where possible respondents were asked to quantify the time spent teaching pain education specifically and whether this involved one-off lectures or complete modules with credit values. This data was then compiled and categorised according to hours of pain education delivery. It was agreed that the public would be blind to students University of study, so that institutional variation is quantifiable but specific institutions could not be directly compared.

### Outcome measures

The survey contained two questionnaires: 1) the 12-item Revised Neurophysiology Questionnaire RNPQ [41] to measure pain knowledge, and 2) the 13-item Health Care Providers Pain and Impairment Relationship Scale HC-PAIRS [42] to measure attitudes towards chronic pain. These questionnaires together were estimated to take less than 10 min to complete.

#### The Revised Neurophysiology of Pain Questionnaire (RNPQ)

This 12-item questionnaire was used to assess knowledge of pain neurophysiology. Responses are marked ‘yes’, ‘no’ or ‘undecided’ the latter being important to prevent respondents from guessing the answer. Scores range from 0–12 with high scores indicating a good knowledge of pain neurophysiology. The RNPQ was developed from the original 19-item Neurophysiology of Pain Test [43]. It was found to have reasonable internal consistency person separation index = 0.84 and good test-rest reliability with an intra-class correlation coefficient value of ICC = 0.97. The RNPQ has now been used consistently in patient, student, clinician and clinical administration staff studies since its inception [44–48]. Furthermore, it is a discipline generic rather than a discipline specific questionnaire, therefore fit for a multi-disciplinary group. There is no established minimally clinically important difference MCID for the RNPQ. However, this can be tentatively estimated as half the baseline SD presented in previous studies [49–52]. Based upon data from Catley et al. (2013) [41] the MCID for RNPQ knowledge was set at 0.9 points or 7.3%.

#### The 13-item modified Health Care Providers Pain and Impairment Relationship scale (HC-PAIRS)

The modified HC-PAIRS [42] measures HCPs’ attitudes towards patients with chronic pain and their functional ability. It features a 7-point Likert scale in 13-items with scores ranging from 13 to 91, the lower score indicates a more positive attitude towards pain. Psychometric properties of the HC-PAIRS are well established. Excellent internal consistency has been demonstrated Cronbach’s α = 0.92 [53] as well as good test–retest reliability [ICC = 0.84] 95% confidence interval 0.78–0.89. Latimer, Maher and Refshauge (2004) [54] also observed its adequate responsiveness to change. Overall, the psychometric properties of the HC-PAIRS are superior to other tools and hence it is consistently widely used [54–57]. A previous study about student HCPs estimated an MCID of 4.5 for the HC-PAIRS [52, 57]. However, Dworkin et al. (2008) [52] advise that MCIDs should be population specific, thus, for this study, the MCID was set at 4.2 points 4.6% based upon half the baseline values for HC-PAIRS data from student HCPs (Colleary et al. 2017) [44]. Originally designed to question attitudes about chronic low back pain Houben et al. (2004) [42] suggest that it is a good measure of chronic pain generically.

### Data analysis

Missing data for the HC-PAIRS was managed as follows: data sets were retained if they were full sets or had only one answer missing [42, 58]. Those with more than one unanswered question were discarded from the data set. Missing answers were replaced with a neutral response, 4 [58]. There are no recommendations within the literature regarding how missing data from the RNPQ should be handled. Thus, for consistency, a similar approach to that of the HC-PAIRS was taken in that a single missing answer in a questionnaire was replaced with a ‘0’ value indicating an incorrect answer. Questionnaires with more than once missing answer were discarded.

Data were analysed using SPSS version 26.0. The data were found to have a normal distribution after a visual inspection of histograms and Q-Q plots, and statistical analysis via the Shapiro–Wilk test. Descriptive statistics are presented as the mean and 1SD. Data were analysed using two-way ANOVA with year of study first or final, and discipline of degree Physiotherapy; Occupational therapy; Nursing; Midwifery; Paramedic; Radiographer as independent variables for the HC-PAIRS and RNPQ separately. The interaction effects of the two independent variables year of study*discipline of degree were also investigated. In addition, a series of post-hoc independent samples t-tests were undertaken to identify where differences lay between individual disciplines and the first and final year of study in each discipline. Correlation analyses were also undertaken as part of a secondary analysis to explore the association between hours of pain education teaching, and knowledge and/or attitude scores, adjusting for age, gender, year of study and discipline. A *p*-value of < 0.05 was considered statistically significant.

## Results

### Response rate

There were 1156 respondents from the 12 universities out of 4067 invitations to participate, representing a 28% response rate. Eight incomplete paper questionnaires were removed for HC-PAIRS six sets and RNPQ two sets as they were almost entirely incomplete. In addition, 162 RPNQ questionnaire data sets were removed as an incorrect version of the questionnaire was accidentally circulated due to human error. This left 1154 respondents who completed and returned surveys adequately, and whose data were analysed. Fifteen of these respondents had left one question unanswered in one of their surveys, nine in the HC-PAIRS questionnaire and eight in RNPQ.

Participants had a mean (SD) age of 26 (8) years, were predominantly female 82% and studying at BSc level 83%. A breakdown of surveys returned can be seen in Table 1, by University and by discipline. Nursing students were categorised together irrespective of speciality as not all respondents disclosed their area of speciality. Some universities returned more surveys than others, and some disciplines had a higher response rate than others, with physiotherapists and nursing students returning the largest numbers of surveys. The overall response rate was lower amongst final year students except in nursing which was heavily dominated by a strong return at one University.

**Table 1:** Number of respondents per University and breakdown of number of respondents in first and final year by discipline

University Code	Number of responses	Disciplines Surveyed	First year respondents	Final year respondents
1	11	**Occupational therapists**	43	34
2	134			
3	8	**Physiotherapists**	266	104
4	514			
5	12	**Paramedics**	68	9
6	126			
7	51	**Midwives**	32	11
8	47			
9	11	**Nurses**	235	312
10	120			
11	97	**Diagnostic radiographers**	31	9
12	23			
**Total**	**1154**	**Total**	**675**	**479**

### HC-PAIRS

The two-way ANOVA for HC-PAIRS found a significant independent effect of both year of study *p* = 0.001 and discipline *p* = 0.001. Table 2 lists the mean HC-PAIRS attitude scores for individual professions. First year mean values ranged from 54.4 to 60.0 lower values indicating more positive attitudes. In final year they ranged from 37.5 to 56.1. Between first and final year the greatest improvement in attitudes to pain was shown by physiotherapy students, with a mean difference 95% confidence interval [CI] of -17.2 [-19.2 to -15.2] points. All of the other professions showed clinically insignificant, less than or equal to the MCID, and statistically insignificant changes from first to final year. This is with the exception of nursing which showed a clinically insignificant but statistically significant improvement -2.2 [3.6 to -0.7] *p* = 0.03. A two-way ANOVA revealed that there was a statistically significant interaction (*p* < 0.01) between the effects of the two independent variables year of study*discipline of degree.

**Table 2:** HC-PAIRS, pain attitude scores for first and final year by profession

Profession *n* = total number	1^st^ Year Mean (SD)	Final Year Mean (SD)	Mean Difference	95% CI	*P*-value
OT *n* = 77	56.4 (8.6)	52.8 (7.3)	-3.7	-7.4 to 0.1	0.51
Physiotherapy *n* = 370	54.7 (8.8)	37.5 (9.1)	-17.2	-19.2 to -15.2	0.01*
Paramedics *n* = 77	55.7 (8.2)	52.1 (8.3)	-3.6	-9.4 to 2.3	0.23
Midwifery *n* = 43	60.0 (9.6)	56.1 (8.6)	-3.9	-10.5 to 2.7	0.24
Nursing *n* = 547	57.1 (8.0)	55.0 (8.5)	-2.2	-3.6 to -0.7	0.03*
Diagnostic Radiography *n* = 40	54.4 (9.0)	51.6 (8.9)	-2.9	-9.7 to 4.1	0.40

*SD* standard deviation, *CI* confidence interval, *HC-PAIRS* Health Care Providers Pain and Impairment Relationship Scale. *P*-values were calculated using independent t-tests

^*^ Indicates statistical significance at *p* < 0.05

**Fig. 1:**
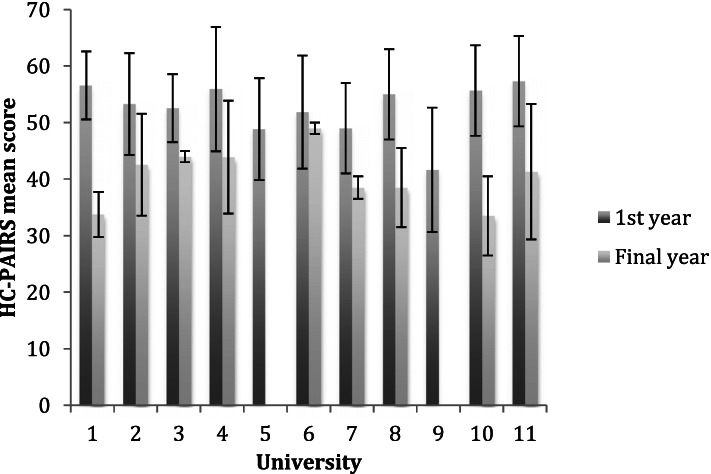
First and final year mean SD HC-PAIRS scores for physiotherapy cohorts in Universities 1–11. University 12 did not include any physiotherapists

As physiotherapy was the only discipline that showed a clinically and statistically significant change from first to final year, secondary analysis was carried out within that discipline to explore if all universities performed equally well as shown in Fig. 1. Seven of the eight universities, which had first and final year respondents, showed a difference between the year groups, exceeding the MCID of -4.2, ranging from -8 to -23 units. University 6 had a mean change of less than -4.2. This may have been an artefact of the very small number of respondents from this sub-group. There were only 17 first year respondents and only two final year respondents thus it was not representative of the final year. Two universities, codes 5 and 9, had only first year participants and not final years; one University did not have any physiotherapy respondents code 12.

### RNPQ

Two-way ANOVA for RNPQ found a significant independent effect of year of study *p* = 0.044 and discipline *p* = 0.025. Table 3 lists the mean RNPQ knowledge scores for individual professions with higher scores indicating better knowledge of pain neurophysiology. The minimum mean (SD) score in the first year was 5.7 (2.0) and the maximum was 7.3 (1.8). Final year scores ranged from a minimum of 5.7 (2.1) and maximum 9.1 (2.0). The biggest improvement in pain knowledge between first and final year is shown by physiotherapy students with a change of 3 points, a difference which was statistically significant *p* = 0.01. All the other professions showed clinically small less than or equal to the MCID and statistically insignificant differences from first to final year. A two-way ANOVA revealed that there was a statistically significant interaction (*p* < 0.01) between the effects of the two independent variables year of study*discipline of degree.

**Table 3:** RNPQ pain knowledge scores for first and final year by profession

Profession Total numbers, *n* =	1^st^ Year Mean (SD)	Final Year Mean (SD)	Mean Difference	95% CI	*P*-value
Occupational Therapy *n* = 77	5.9 (1.8)	6.4 (1.6)	0.5	0.3 to 1.3	0.26
Physiotherapy *n* = 370	5.7 (2.0)	9.1 (2.0)	3.4	3.0 to 3.9	0.01*
Paramedics *n* = 77	6.1 (1.5)	5.7 (2.1)	-0.4	-0.9 to 1.8	0.48
Midwifery *n* = 43	6.1 (2.0)	7.00 (1.4)	0.9	0.6 to 2.3	0.24
Nursing *n* = 547	5.9 (2.0)	6.2 (2.0)	0.3	0.1 to 0.7	0.06
Diagnostic Radiography *n* = 40	7.3 (1.8)	6.0 (2.1)	-1.3	-0.4 to 3.0	0.13

*RNPQ* revised Neurophysiology Questionnaire, *SD* standard deviation, *CI* confidence interval; *P*-values were calculated using independent t-tests

^*^ Indicates statistical significance at *p* < 0.05

**Fig. 2:**
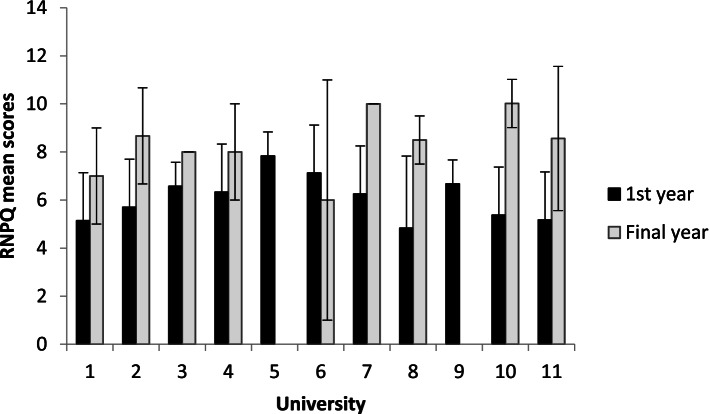
First and final year mean RNPQ scores for physiotherapy cohorts in Universities 1–11. University 12 did not include any physiotherapists

Once again, as they were the only discipline to have demonstrated a statistical and clinical difference between first and final year cohorts, secondary analysis of the physiotherapy data were carried out to explore if some universities made greater gains than others. The minimum mean difference was 1.1 95%CI [2.9 to 5.2] and the maximum mean difference was 4.7 [4.0 to 5.3] see Fig. 2. Thus, the size of pain knowledge improvement was not consistently high in all physiotherapy cohorts at all of the universities sampled, but always exceeded the MCID of 0.9 points.

### Secondary analysis

Multiple linear regression analyses were completed to explore the association between hours of pain education in all of the disciplines studied, and knowledge and attitude scores respectively, adjusting for age, gender, year of study and discipline.

For both dependent variables, pain knowledge and pain attitudes, hours of pain education teaching was found to be an independent predictor though the strength of the relationship was small (RNPQ ß value = 0.11, *p* = 0.01 and HC-PAIRS ß value = 0.15, *p* = 0.001).

**Fig. 3:**
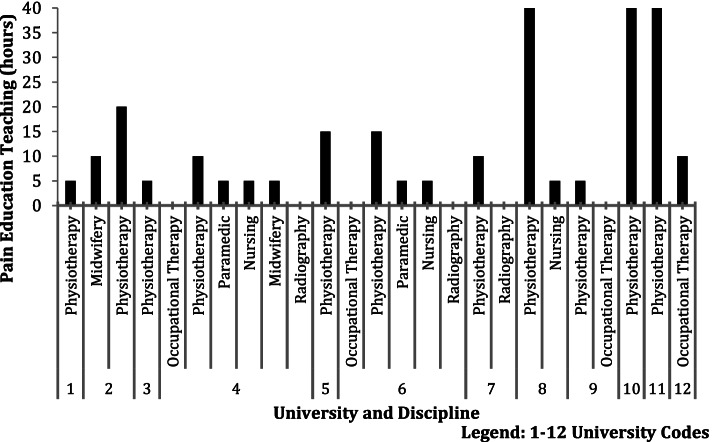
Approximate hours of pain education teaching in each discipline and University

The amount of focused pain teaching at the time of data collection varied considerably between universities and disciplines. Figure 3 reflects this difference with physiotherapy departments generally delivering the greatest amounts of pain education teaching.

## Discussion

There has been recent suggestion that there is a need to shift understanding about pain on a societal level in order to optimise and contemporise care [59]. HCPs will be a key sector of society to focus upon as they will influence the pain understanding of others. Furthermore, targeting HCP students, whose understanding may be more malleable, may be the optimal point at which to target HCPs. An important step in this process is to survey pain attitudes and knowledge amongst future health care workers to quantify current levels of understanding and identify if training could be enhanced. Accordingly, this study compared the pain knowledge and attitudes in first and final year students, across six disciplines, at 12 institutions, in five countries. To date, this is the largest, international cross-sectional study to quantify the knowledge and attitudes about pain amongst NMAHP students. There were differences in pain knowledge and attitudes between year of study and between disciplines. There was also a year of study*discipline interaction effect. Of the six disciplines, physiotherapy had the greatest mean differences between the first and final year for both the RNPQ and the HC-PAIRS which were clinically and statistically significant. In contrast there was little difference between first and final year values for both knowledge and attitudes scores in the other disciplines.

The nursing cohorts showed the least improvement in attitudes with a mean difference of -2.2, well below the MCID of 4.2 of all disciplines, yet statistical testing showed the difference to be significant *p* = 0.03. It is likely that this was due to the larger sample size for the nursing group and thus greater statistical power. However, the magnitude of the difference is well below the MCID and thus likely to be clinically unimportant.

Direct comparison with existing literature is difficult as a large portion of the literature uses different outcome measures, and studies using similar outcome measures include physiotherapy students only. The improvement in attitudes for physiotherapy students over the duration of a degree programme, as measured by the HC-PAIRS, in this study are greater than previously reported [37, 60, 61], but scores were not quite as high as the changes measured in RCTs following targeted, brief pain science education interventions directly addressing knowledge and attitudes in physiotherapists [44] and NMAHPs [48]. This suggests there is scope for greater changes on the observed degree programmes in this study.

Whilst Carroll et al*.*, (2020) [40] found greater improvement amongst their nursing cohorts’ attitudes (1.6%—7% amongst different nursing specialities) than in this study, 2.4%, our findings accord with Amponsah et al*.* (2020) [62] and Leahy et al*.* (2019) [63] that final year nurses have considerable deficits in pain knowledge and attitudes. Mukoka, Olivier and Ravat (2019) [64] found more positive attitudes in their nursing and occupational therapy students but not as positive among their physiotherapy students. Overall the findings from this study generally concur with the existing body of literature that suggests there is a deficiency in pain knowledge and attitudes towards pain in final year HCP students. Many previous studies noted an improvement in HCP students’ knowledge and attitudes from first to final year [37, 38, 62, 64, 65], and while we found this among physiotherapy students, it was not the case overall. Worryingly, Ryan et al*.*, (2010) [61] noted that non-health care students demonstrated a 3.9 point 3.7% mean difference in HC-PAIRS 15-point questionnaire from first to final year. This is similar if not better than the difference seen for the health care students in the current study, apart from physiotherapy students. The comparatively poor difference in pain attitudes demonstrated for most disciplines other than physiotherapy in this study may be attributable in part to a biomedical model-based curricula impeding the natural small biopsychosocial shift with time seen in the non-health care programme sample studied by Ryan et al. (2010) [61].

There were larger volumes of pain specific teaching on the physiotherapy courses relative to the other NMAHP disciplines in the current study (Fig. 3). This is perhaps unsurprising as physiotherapists may be perceived to play a larger role in pain management than some of the other disciplines. The larger differences between first and final year in physiotherapy are likely in part due to the higher volumes of pain specific teaching. Within our data, there was a moderate/high correlation between difference in attitudes and knowledge and higher volumes of pain teaching *r* = *0.5, p* = *0.16* and *r* = *0.7, p* = *0.03* respectively. This provides a rationale for larger volumes of pain teaching within NMAHP curricula.

An additional factor influencing student pain knowledge and attitudes that has not been explored in this study is the effect of clinical placements. This aspect of health care education warrants further investigation as it may positively or negatively [66] influence pain management behaviours.

Thompson et al*.* (2018) [27] propose an array of reasons that inhibit the implementation of effective pain education into pre-registration health care programs. These authors suggest that all health care disciplines have different curricula pressures placed upon them by internal and external bodies, and pain education may not yet be recognised as a priority topic for these health care disciplines. Furthermore, professional opportunities to manage pain are not always the focus of some disciplines and some disciplines may play a larger role in the care pathway than others and thus arguably may need higher levels of knowledge and attitudes relative to other disciplines. However, each discipline involved in this study may encounter people with pain directly and as such it is important that they all have appropriate knowledge and attitudes to provide patients with clear and consistent high quality basic pain management advice For example, in diagnostic radiography patient interaction may be limited, nevertheless, even if interactions are brief, correct communication is critical [67, 68]. Kyei et al. (2014) [69] observe the need for good radiographer communication skills because there is only a short time frame available to establish a relationship with patients. Furthermore, the reports that an extended scope radiographer may be required to complete are often shown to patients and it is important that these report any anomalies within the context of age-related changes and the possibility that an individual’s pain may not always be linked to the findings [70–73]. Ultimately, failures from a key team member in a pain management multi-disciplinary team can affect the pain management efforts of the whole team and thus patient outcome.

### Limitations

The observational, cross-sectional nature of this study means that no claim of cause and effect can be made. Measuring students in the first and final year meant it was impossible to identify at what points in training pain knowledge and attitudes changed, and thus understand what aspects of training may influence change. Future studies should employ a longitudinal design, measuring students yearly to identify potential triggers for improving knowledge and attitudes towards pain, taking into account student placements and their impact. In addition, a longitudinal study would help to establish if the cross-sectional differences seen in this study are comparable to changes in the same cohort of students followed over the course of their degree. There is a need for pain management behaviours resulting from education to be investigated specifically, though changes in knowledge and attitudes can be predictors of behaviour [30].

Some universities and disciplines returned more responses than others, thus there may be a response bias in this snapshot of pain knowledge and attitudes in students.

There was not an *apriori* sample size calculation. Instead, the researchers attempted to recruit as many participants as possible from the institutions involved. As such it is possible that the study is underpowered for some disciplines and may explain the lack of statistical differences between first and final year students for some disciplines. However, the magnitude of the differences between first and final year, would be less likely to be influenced by sample size and those differences were small and well below the MCID for all except the physiotherapy group.

In a small minority of cases the number of participants in sub-groups were very small. In such cases the sub-analysis was exploratory and should be interpreted with caution.

The differing sample sizes may have been due, in part, to final year students being on clinical placements at different times, and thus being less receptive to email invitations to participate in this study. Other factors may have been survey fatigue; the National Students Survey NSS was underway in the England, Scotland and Northern Ireland at a similar time to data collection, as well as individual module feedback surveys at many universities. Despite this, every attempt was made to access final year students at the end of their degree programme, including extending the study for a further year of data collection.

Participant self-selection may have influenced sample size. The pain champion at each of the universities may not have equally reflected all disciplines. The majority of pain champions were physiotherapists. This may have accounted for the larger numbers of physiotherapists relative to other disciplines for example only two universities represented paramedic training whilst 11 universities represented physiotherapy. Arguably medical doctors, such as general practitioners GPs and anaesthesiologists, will have more involvement in pain management than some NMAHPs and it would be illuminating to include this health care discipline in future studies of student knowledge and/or attitudes.

In one quarter of the physiotherapy courses investigated there was up to 40 h of pain education teaching and this is reflected in the difference in knowledge and attitudes in first and final year physiotherapy students. This volume of teaching may not be reflective of all physiotherapy courses, and may inflate the overall variance between disciplines. Furthermore, the time spent teaching pain education is of interest, but the content of that education is also important [74]. This study did not investigate the content of pain education being delivered and future studies should investigate the impact of educational content on pain related knowledge and attitudes.

## Conclusions

To date, this is the largest investigation of HCP student pain related knowledge and attitudes amongst NMAHPs, including 12 universities and six disciplines in five countries. Only physiotherapy students showed statistically and clinically significant improvements in pain related attitudes and knowledge from first to final year. The differences were correlated with the volume of pain teaching received. Given that clinicians with more positive attitudes towards pain are more likely to make evidence-based recommendations, in turn improving patient outcomes, this study highlights the need to improve NMAHP pain education.

## Data Availability

The datasets used and/or analysed during the current study are available from the corresponding author on reasonable request.

## Abbreviations

ANOVA: Analysis of variance
AUD: Australian Dollar
BSc: Bachelor of Science
CI: Confidence Interval
GBD: Global Burden of Disability
GP: General practitioner
HCP: Health Care Professionals
HC-PAIRS: Health Care Providers Pain and Impairment Relationship Scale
IASP: International Association for the Study of Pain
MCID: Minimally clinically important difference
N: Number
NMAHP: Nursing, Midwifery and Allied Health Professionals
NSS: National Student Survey
PRIME: Prevalence Impact and Cost of Chronic Pain
RNPQ: Revised Neuro Physiology Questionnaire
SD: Standard deviation
SPSS: Statistical Package for the Social Sciences
TU: Teesside University
UK: United Kingdom
